# Survival and storage stability of encapsulated probiotic under simulated digestion conditions and on dried apple snacks

**DOI:** 10.1002/fsn3.1815

**Published:** 2020-08-25

**Authors:** Muhammad Afzaal, Farhan Saeed, Shahzad Hussain, Abdellatif A. Mohamed, Mohamed S. Alamri, Aftab Ahmad, Huda Ateeq, Tabussam Tufail, Muzammil Hussain

**Affiliations:** ^1^ Institute of Home & Food Sciences Government College University Faisalabad Faisalabad Pakistan; ^2^ Department of Food Science & Nutrition King Saud University Riyadh Saudi Arabia; ^3^ The University of Gambia Serrekunda Gambia

**Keywords:** apple snack, probiotic viability, probiotics, simulated conditions, storage stability

## Abstract

The objective of the current study was to explore the probiotics carrier potential of apple dried snacks and improve the survival of probiotics under simulated gastrointestinal conditions. Purposely, the probiotics were encapsulated using two hydrogel materials (sodium alginate and carrageenan) by using encapsulator. Briefly, slices of apple were immersed in solution containing free and encapsulated probiotics and then dried by conventional drying method. The dried apple snack was analyzed for different characteristics (physiochemical and microbiological) during storage. The viability of the free and encapsulated probiotics was accessed in apple snack and under simulated gastrointestinal conditions. Apple snack rich with encapsulated probiotics showed a significant result (*p* < .05) regarding the survival and stability. The encapsulated probiotics decreased from 9.5 log CFU/g to 8.83 log CFU/g as compared to free probiotics that decreased to 5.28 log CFU/g. Furthermore, encapsulated probiotics exhibited a better stability under simulated gastrointestinal conditions as compared to free. During storage, an increase in phenolic content and hardness was observed while decrease in pH was noted. Results of sensory parameters indicated apple snack as potential and acceptable probiotics carrier.

## Graphical abstract

The current research is helpful in broadening the spectrum of encapsulation application for the development of nonfermented product as probiotic carrier. Microencapsulation is trending technique to improve the bioavailability of probiotics under different stressed conditions. Encapsulation ensured therapeutic level (107–108 cfu/g) of probiotics in carrier food as well as under simulated gastrointestinal conditions. The experimental work is scientific basis for the stakeholders to utilize for product development at commercial level.

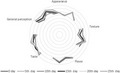

## 1 INTRODUCTION

A snack is a product which is easy to consume and needs no preparation before consumption. It can be used to satisfy the hunger between meal times (Herman, Polivy, Pliner, & Vartanian, 2019). People have been consuming fried snack products for years due to attractive aroma, flavor combinations, and texture. However, owing to their certain health hazards, there is an increasing interest among people for consumption of safe, healthy, and nutritious food (Santeramo et al., 2018). This has led to an emphasis on the production of food free from extra calories, sugars, and fat. Only a small number of crunchy snacks that can be considered healthy are available in the market nowadays. An exciting alternative to currently popular snacks is dried apple snacks.

The use of vegetables and fruits in every day diet has led an increase in health benefits such as fewer calories, more dietary fiber and fiber content, and numerous nutritive components that include minerals and vitamins (Higgs, Liu, Collins, & Thomas, 2019). In a report by WHO, it has been recommended that fruits and vegetables other than starchy tubers and potatoes help in retaining a healthy life style and prevent a number of diseases like diabetes, cancer, heart problems, and obesity. The consumption of fruits and vegetables also helps to meet certain deficiencies of nutrients in the body as well (WHO, 2004).

Along with the fresh fruits, they can be taken in frozen, juice, canned, or dried form as well (Keast & Jones, 2011). Among fruits, apples are considered as a healthy diet owing to good nutritional composition with high fiber and polyphenolic contents. The presence of high phenolic contents is helpful in prevention of different chronic diseases. These phenolic contents have potential anticarcinogenic and anti‐inflammatory activities (Boyer & Liu, 2004; Gardner, White, McPhail, & Duthie, 2000; Lee, Kim, Kim, Lee, & Lee, 2003; Sun, Melton, O’Connor, Kilmartin, & Smith, 2008). Drying being the oldest food preservation method has an extensive use over centuries. Drying not only prolongs the shelf life of fruits and vegetables, it also lowers the water activity and prevent microbial or enzymatic activity and also increases their nutritional properties, total energy, nutrient density, fiber content, and greater antioxidant activity due to removal of extra water (Doymaz, Karasu, & Baslar, 2016).

Dried food consumption has also been linked with better nutrient consumptions, higher overall diet quality scores, and lesser body weight/adiposity measures (Keast et al., 2011). An important way of consuming healthy foods is to include probiotics in the diet. Probiotics are defined as living microorganisms that are beneficial for human body when consumed at sufficient levels (FAO, 2006; Hill et al., 2014). Consistent intake of viable probiotics can confer countless health benefits such as a antidiabetic properties (Devalaraja, Jain, & Yadav, 2011), improvement of lactose tolerance (Shah, 2007), and reduction of cholesterol (Nguyen, Kang, & Lee, 2007). Numerous efforts have been made for manufacture of probiotic enriched dried fruits and fermented probiotic fruit juices (Rein et al., 2013).

To achieve the most beneficial effects at target sites, probiotics reflect resistance toward hostile conditions in gastrointestinal tract (Rokka & Rantama, 2010). Protection of probiotics by microencapsulation improves their viability under adverse conditions in gastric conditions. Microencapsulation improves the resistance and survival of probiotics under various food processing conditions and can raise the probiotics level in human digestive system. Among other potential ingredients, alginate biopolymer is the most studied and examined material for encapsulation of probiotics (Zanjani, Ehsani, Tarzi, & Sharifan, 2018). Sodium alginate as a natural biopolymer has extensive application in encapsulation purpose owing to different attributes including economical, on‐toxic, simplicity, biocompatibility, and biodegradability. Sodium alginate polymer has porous protective structure that help in diffusion (Chandy, Mooradian, & Rao, 1998; Devi & Kakati, 2013; Islan, De Verti, Marchetti, & Castro, 2012). Both sodium alginate and carrageen wall materials are economical, nontoxic, and easy to handle (Liu, Xie, & Nie, 2020).

The core objective of the present study was to prepare dried apple‐based innovative probiotic enriched snack with the addition of *Bifidobacterium bifidum*. The prepared product was analyzed for microbiological, physicochemical, and sensorial attributes over a period of 25 days of storage.

## 2 MATERIALS AND METHODS

Fresh apples (*Malus domestica*) of good quality (Kala kulu) were purchased from Faisalabad Market. Apples were selected on the basis of their size, color, and absence of any physical harm. Probiotic Culture (*Bifidobacterium bifidum)* was obtained from NIFSAT, University of Agriculture Faisalabad. All general chemicals (Sigma‐Aldrich GmbH, Sternheim, Germany) were purchased from scientific store. The research was carried out at Food Safety and Biotechnology laboratory, Government College University, Faisalabad. Sodium alginate having ratio of mannuronic (M) to guluronic (G) units (M/G = 65/35) and molar weight (MW = 90–180 KDa) was used in this study.

### 2.1 Culture activation

Pure freeze‐dried cells were activated by inoculating it in MRS (Man Rogosa Sharpe) broth for 24 hr at 37°C. Afterward, the cells were centrifuged in a centrifuge machine (750073276 EA, Thermo Fisher Scientific Inc. USA). The obtained probiotic cells were encapsulated as described below.

### 2.2 Encapsulation process

The probiotic bacteria *B. bifidum* was encapsulated with k‐carrageenan and sodium alginate microgels by following the method as described by Afzaal et al. (2018) with little modifications. Shortly, 100 ml of 3% (w/v) k‐carrageenan (KC) and sodium alginate (SA) solutions wase prepared. The prepared solutions were autoclaved at 121oC [15psi] for 15 min. The aseptic solutions of sodium alginate and carrageenan were mixed with 2 ml of 10^10^ cfu/ml probiotics obtained in activation process. The beads were prepared aseptically by using an encapsulator [B‐390, Buchi‐Switzerland] in standard operating conditions. Encapsulating materials liquors were injected into hardening solutions of calcium chloride (0.1 M) solution. Later on, the desired beads were acquired by filtration, washed by sterile deionized water. The acquired beads were stored in physiological saline solution at 4°C.

### 2.3 Encapsulation yield

The encapsulation yield was calculated by using the method of Iqbal, Zahoor, Huma, Jamil, and Ünlü (2019). For this purpose, 20 microbeads were selected randomly from both type of encapsulated formulations. The selected beads were disintegrated using a stomacher bag containing a phosphate buffer solution and a solution of sodium citrate having a molarity of 0.1 M at pH of 6.3 by using a stomacher bag. The number of released cells from each type (‐Sodium Alginate and kappa‐ carrageenan) was determined by using pour plate technique. The yield was calculated by using the following formula:

$$\text{EY}=\frac{\text{Number of cells released (sodium alginate bead and k}‐\text{carrageen bead)}}{\text{Number of cells added (sodium alginate and k}‐\text{carrageenan solution)}}\times 100.$$

### 2.4 Sample preparation

Apples were first washed with tap water, and all the dirt was removed by scrubbing it gently. After washing, the peel was removed with the help of a peeler and apples were cut in to disk‐shaped slices (having diameter 15 mm and thickness 6 mm). For preventing apple slices from enzymatic browning throughout storage and drying, the slices of apple were heated in water bath at 80°C for 2–3 min.

### 2.5 Probiotic Inoculation in apple slices

As *B. bifidum* was used as a probiotic which was inoculated in apple slices by following the method of Akman, Uysal, Ozkaya, Tornuk, and Durak (2019), with a slight modification. Inoculum in the range of 9–10 log CFU/g was added in peptone water (∼1%), and slices of apples were dipped in a solution (1:3 apple/liquid ratio w/v) for 8 min at an ambient temperature with moderate stirring. Afterward, the apple samples were kept in controlled environment for 15–20 min at ambient temperature to enable the probiotics attachment on apple slices. Control treatment was dipped in disinfected peptone water to avoid it from adverse environment conditions, without adding bacterial culture. Treatment plan for this research work is shown below (Table 1).

**Table 1:** Treatment plan for the development of apple snack

Sr no.	treatments	Details
1	ASC	Dried apple snack (control)
2	ASWFP	Apple snack with free probiotics
3	ASWSA	Apple snack with probiotics encapsulated in Sodium alginate
4	ASWKC	Apple snack with probiotics encapsulated in K‐carrageenan

### 2.6 Drying of apple slices

Apple slices were dried using conventional method of drying. Drying was done by using an oven dryer at 40–50°C until the moisture content left less than ≤12%. Normally, the drying interval was about 6h, respectively. After this drying process, the apple slices were packed in a closed polyethylene cups that were stored at 4°C for 25 days. All prepared snacks were evaluated after an interval of 5 days.

### 2.7 Enumeration of *B. bifidum*

For enumeration of *B. bifidum*, 10 g slices of apple were assimilated with 90 ml of sterilized peptone water and standardized with a help a stomacher at an average speed for 2 min. serial dilution of ten‐fold were prepared from the normalized samples with peptone water. Dilutions were inoculated in suitable form onto MRS Agar (Merck, Germany), and petri dishes were incubated for 48 h at 37°C. Afterward, colonies of *B. bifidum* were enumerated (CFU/g).

### 2.8 Determination of physicochemical properties

pH of the apple slices was determined by following AOAC (2006) method. pH was measured by a digital pH meter, and readings were noted as a mean of three replicates. The snack from each treatment was added in distilled water, and the pH was determined.

The value for dried apple slice hardness was determined as stress at the peak force. The stress at the peak force is associated with the hardness of the dried slices of apple. The experiments were performed by a texture analyzer. Experiments were performed in three replications, and the mean value was noted in grams (Albertos et al., 2016).

### 2.9 Determination of phenolic components

The phenolic contents of all treatments were determined to investigate the impact of free and encapsulated probiotics enriched apple snacks. All samples were minced by using mixer to determine the total phenolic contents (TPC) in all dried apple snacks. Briefly, 2 g apple slices were combined with 35 ml of 85% methanol (v/v) and stirred at a temperature 37°C with the help of a normal mechanic stirrer at 160 rpm per minute. The extracts of methanol compounds were acquired by the filtration process. Now for extraction of phenolic content of dried apple snacks, the method of Akman et al. (2019) was followed with some modifications. Shortly, 1 ml of the prepared methanolic extract was homogenized with 3 ml of 0.2 N Folin–Ciocalteu's phenol reagent, which was then incubated for 5 min, then 2 ml of 6% Na_2_CO_3_ was poured in it. The obtained solution was kept at for 30 min in dim light at an ambient temperature. Afterward, the samples were measured for absorbance at 760 nm using a spectrophotometer (Shimadzu, U‐1510, Japan) and numerical data were recorded. The total phenolic was expressed as mg gallic acid equivalents per gram dry weight (mg GAE/g DW).

### 2.10 In vitro gastrointestinal assay

In vitro studies were carried out to investigate the survival of free and encapsulated probiotic bacteria in simulated gastrointestinal conditions. Purposely, simulated solutions were prepared by using analytical grade chemicals and aseptic conditions.

### 2.11 Survival of free and encapsulated probiotic bacteria [*Lactobacillus acidophilus*] in simulated gastric juice [SGJ]

Free and encapsulated beads of sodium alginate and carrageenan were subjected to simulated gastric fluid/juice. The tolerance of free and encapsulated probiotic bacteria was determined by following method of Ahmed, Mudgil, and Maqsood (2019) with some modifications. The simulated gastric fluid was prepared by adding pepsin (3 g/L) in sterile sodium chloride solution. The pH of the prepared gastric juice was adjusted by using 0.1 N HCl. The pH of the solution was adjusted to about 2. The prepared solution was stored for the experiment. One gram of microbeads was suspended in a prepared simulated gastric solution. Free/unencapsulated and encapsulated probiotic bacteria were mixed with the gastric solution and were incubated in shaking incubator at 37°C at 110 rpm. MRS agar was used to determine the number of viable bacteria by counting the CFU. The survivability of probiotic in free and encapsulated was recorded with a time interval of 0, 30, 60, 90, and 120 min. The results were recorded in triplicate.

### 2.12 Survival of free and encapsulated probiotic bacteria in simulated intestinal juice [SIJ]

The survival of probiotics is also important in the intestinal conditions after their passage through the stomach. The survivability of free and encapsulated probiotic bacteria was accessed by the method as described by Nogueira and Grosso (2017) and Singh, Sharma, Chauhan, and Goel (2019). The tolerance of encapsulated bacteria [bead cells] to simulated intestinal transit conditions was determined by subjecting the free and encapsulated probiotic bacteria to the simulated intestinal solution. Briefly, bile salt (3 g/L) and pancreatic (10 g/L) were dissolved in phosphate buffer having pH (8.0). The pH (7.5) of the intestinal fluid was adjusted by using sterilized NaOH. One gram of microbeads was suspended in a prepared simulated gastric solution. MRS agar was used to determine the number of viable bacteria by counting the CFU. Both types of cells, that is, unencapsulated/free and encapsulated, were separately added to prepared simulated solutions and stored in shaking incubator at 37°C and 110 rpm. The viability/survival of free/unencapsulated and encapsulated suspending cells was calculated on pleating MRS agar with encoded interval of time [0, 30, 60, 90, and 120 min].

### 2.13 Sensory evaluation

Evaluation of sensory parameters was achieved using nine point‐hedonic scale with 15 inexpert panelists including 5 females and 10 males between 20 and 30 years of age. The volunteers examined the appearance, texture, flavor, taste, and general perception of the treatments by scoring from 1 to 9 (9 points, extremely good and 1 being extremely poor). All snacks samples (coded) were randomly presented to the panel for evaluation.

### 2.14 Statistical analysis

Standard deviations and mean results of the values were obtained using SPSS software. Statistical examination was done by software Statix‐8 with analysis of variance (ANOVA). Difference between the data was estimated by means of LSD and multiple comparison tests at a significance 95% level.

## 3 RESULTS AND DISCUSSIONS

### 3.1 Encapsulation yield

Constituents of wall medium have a direct effect on the yield of encapsulation. The encapsulation yields also affect the survival in simulated digestive conditions (Bora, Li, Zhu, & Du, 2018). Both encapsulating materials showed a difference in encapsulation yield. Alginate coating gave a higher yield as compared to carrageenan coating as shown in Table 2.

**Table 2:** Encapsulation efficiency

Type of materials	Number before encapsulation	Number after encapsulation	Efficiency (%)
Sodium alginate (encapsulated)	8.96 ± 0.04	8.79 ± 0.05	98%
K‐Carrageenan (encapsulated)	8.25 ± 0.02	7.77 ± 0.07	94%

Microbeads with wall material sodium alginate showed efficiency (98%) as compared to carrageen as encapsulating efficiency (94%). The type of hydrogel matrices and method of encapsulation directly affect the encapsulation yield (Kavitake, Kandasamy, Devi, & Shetty, 2018). In most of the studies carried out by different scientists proved that sodium alginate is more effective encapsulating agents as compared to other polymer. The results showed that required cell number was entrapped by both type of materials. However, a greater number of cells were entrapped in sodium alginate matrix as compared to carrageenan. An effective intake (10^8^–10^9^ CFU/g) of the product containing probiotics is required for colonizing of the probiotics in the colon. Encapsulation with alginate and whey protein enhanced the survival of probiotics (Chotiko & Sathivel, 2016; Xu, Gagné‐Bourque, Dumont, & Jabaji, 2016).

### 3.2 pH of dried apple snack

**Figure 1:**
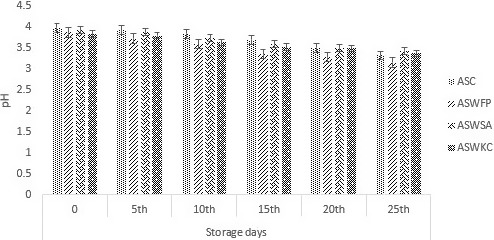
Effect of free (unencapsulated) and encapsulated (with sodium alginate and carrageenan) *Bifidobacterium bifidum* on pH of dried apple snacks during storage intervals (0, 5, 10, 15, 20, and 25 days) compared with control. Each bar represents mean value for pH of treatments. ASC (Control without addition of probiotics), ASWFP (Free/unencapsulated cells), ASWSA (Probiotics encapsulated with sodium alginate), and ASWKC (Probiotics encapsulated with K‐carrageenan)

Figure 1 shows the results regarding pH of all dried apple snack. The study showed that pH was maximum in treatment ASWSA (dried apple snack encapsulated with sodium alginate); however, ASWFP (dried apple snacks with free probiotics) showed the lowest change in pH. This decrease in pH may due to the reason that probiotics may utilize the carbohydrates and produce acids (organic acids and lactic acid). Reduction in the pH due to the inoculation of *B. animalis* ssp.* lactis* in yogurt was suggested by Senadeera et al. in, 2018. Similarly, reduction in the pH of carrot juice was observed due to the addition of *Lactobacillus acidophilus*  by Shigematsu et al., 2018.

### 3.3 Measurement of hardness of dried apple snack

**Figure 2:**
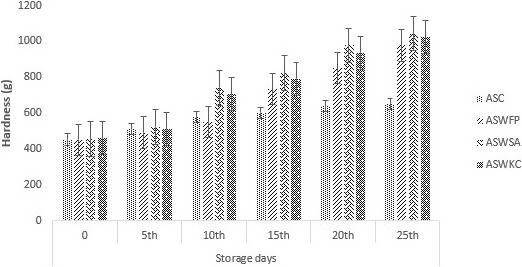
Effect of free (unencapsulated) and encapsulated (with sodium alginate and carrageenan) *Bifidobacterium bifidum* on hardness of dried apple snacks during storage intervals (0, 5, 10, 15, 20, and 25 days) compared with control. Each bar represents mean value for hardness of treatments. ASC (Control without addition of probiotics), ASWFP (Free/unencapsulated cells), ASWSA (Probiotics encapsulated with sodium alginate), and ASWKC (Probiotics encapsulated with K‐carrageenan)

Hardness and permeable configuration are frequently used for the degree of crispness. Among the quality features of snack food, texture is an important factor. For snack, crispness is a well‐known important parameter. The hardness of the ASWSA (dried apple snack encapsulated with sodium alginate) was significantly higher (*p* < .05)* as* compared to all other samples which was shown in Figure 2, possibly because little damage was induced in apple, resulting in an integrated but very thin cell wall. This thin cell wall had a poor capability to repel external factors, leading to reduced hardness (Jiang et al., 2017).

### 3.4 Phenolic components in dried apple snack

**Figure 3:**
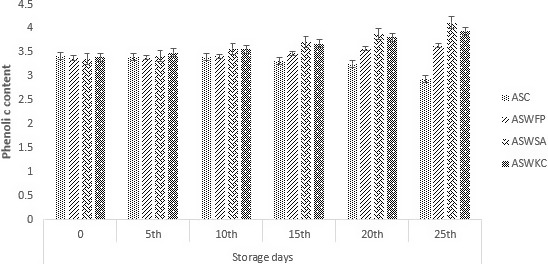
Effect of free (unencapsulated) and encapsulated (with sodium alginate and carrageenan) *Bifidobacterium bifidum* on phenolic contents of dried apple snacks during storage intervals (0, 5, 10, 15, 20, and 25 days) compared with control. Each bar represents mean value for phenolic contents of treatments. ASC (Control without addition of probiotics), ASWFP (Free/unencapsulated cells), ASWSA (Probiotics encapsulated with sodium alginate), and ASWKC (Probiotics encapsulated with K‐carrageenan)

Figure 3 shows the phenolic content of dried apple snack treatments augmented with *B. bifidum*. The initial phenolic content of all the treatments was approximately 3.40 mg GAE/g of the dray material. Results suggested that the phenolic content in the dried apple snack increases with the probiotic addition significantly (*p* < .05). Initial storage days showed no significant (*p* < .05) changes in the phenolic content. Joshi, Rupasinghe, and Khanizadeh (2011) suggested same results in their study. However, after 2 weeks a rapid increase was noted in the phenolic content of the dried apple snack treatments. The maximum value for phenolic components was observed in ASWSA (dried apple snack with sodium alginate coating), while the lowest phenolic content was observed in ASC (control treatment). Pereirain , Almeida, de Jesus, da Costa, & Rodrigues, (2013) proposed that the presence of free probiotics can degrade the phenolic contents, and this might be the reason that dried apple snack with encapsulated probiotics have maximum phenolic contents.

### 3.5 Probiotic viability in dried apple snack

**Figure 4:**
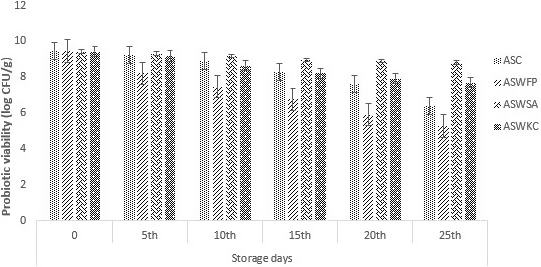
Survival of free and encapsulated (with sodium alginate and carrageenan) probiotics in dried apple snacks during storage intervals (0, 5, 10, 15, 20, and 25 days) compared with control. Each bar represents mean value for survival of treatments. ASC (Control without addition of probiotics), ASWFP (Free/unencapsulated cells), ASWSA (Probiotics encapsulated with sodium alginate), and ASWKC (Probiotics encapsulated with K‐carrageenan)

In the present study, dried apple slice snack was prepared by the addition *B. bifidum* dried by conventional drying method by using dying oven. As apples were kept in water bath with the intention of prevention of enzymatic browning, some microflora was deactivated due to heat. Figure 4 showed the initial microbial content of the probiotics was approximately 9.5 log CFU/g. The survival of probiotics is very important when subjected to different processing conditions. The highest probiotic population was observed in ASWSA treatment with 8.83 log CFU/g probiotics after 25 days of storage. A log reduction of 0.59 CFU/g was observed in this treatment. However, the least probiotic stability of 5.28 log CFU/g was observed in ASWFP (apple with free probiotics) after storage for 25 days. A log reduction of 4.19 CFU/g was observed. These results indicated that probiotic bacteria *B. bifidum* showed a better attachment and viability in encapsulated form with alginate as a coating material. A study was conducted by Romano et al. in, 2014 with methylcellulose edible coating was prepared and *Lactobacillus delbrueckii* subsp. *Bulgaricus* showed better survival.

Another study by Quiroz et al. was conducted in 2014 on green apple baked snack in which encapsulated *Lactobacillus plantarum* was added as a probiotic microorganism which showed significant levels of probiotics after 90 days of storage study. Similarly, a research work was conducted *by* Li et al. (2018), in which probiotic enriched apple snacks were prepared by the addition of *Lactobacillus plantarum* and results confirmed the better viability of bacteria in the final product.

### 3.6 In vitro gastrointestinal tolerance assay

#### 3.6.1 Survival of the encapsulated probiotic bacteria in simulated gastric fluid/Juice

**Figure 5:**
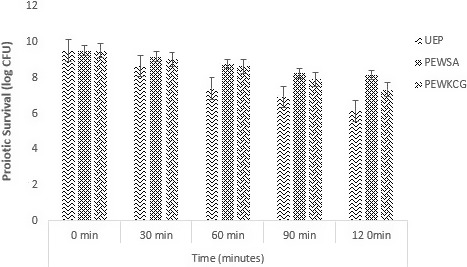
Viability of free and encapsulated (with sodium alginate and carrageenan) probiotics under simulated gastric conditions during storage intervals (0, 30, 60, 90, and 120 min) compared with control. Each bar represents mean value for viability of treatments. UEP (Unencapsulated cells), PEWSA (Probiotics encapsulated with sodium alginate), and PEWKCG (Probiotics encapsulated with K‐carrageenan)

The viability and stability of free and encapsulated probiotics were assessed in simulated conditions. The results showed an instant decrease in case of free cells in contrast to the cells encapsulated with sodium alginate and carrageenan (Figure 5). A rapid reduction was observed in nonencapsulated bacterial cells. A log reduction of 3.37 CFU/g was observed in nonencapsulated or free probiotic cells. However, only 1.36 log reduction CFU/g was observed in encapsulated cells having sodium alginate as a coating material while, and a log reduction of 2.19 CFU/g was observed in case of encapsulated cells with k‐carrageenan as an encapsulation material. Cell viability in stomach and intestinal conditions is important in order to get the desired benefits of probiotics. The results of the present study are in accordance with several authors (Atia, Gomaa, Fernandez, Subirade, & Fliss, 2017; Shi et al., 2013) who demonstrated that use of polymer for encapsulation of probiotics protects and maintain the desired viability of probiotics under acidic environment. The encapsulation of the cells with hydrogel materials improves the viability and stability in low pH medium. The survival rate of probiotics in case of sodium alginate was higher as compared to carrageenan. Poor survival in case of unencapsulated cells was recorded. The results demonstrated that encapsulation provides protection to probiotics in simulated gastric conditions. The results defined that encapsulation of cells showed a shielding result toward probiotics in simulated gastric conditions. Similar results were also obtained by Afzaal et al. (2019), who reported a poor survival of free probiotics.

#### 3.6.2 Survival of the encapsulated probiotic bacteria in simulated Intestinal Conditions

**Figure 6:**
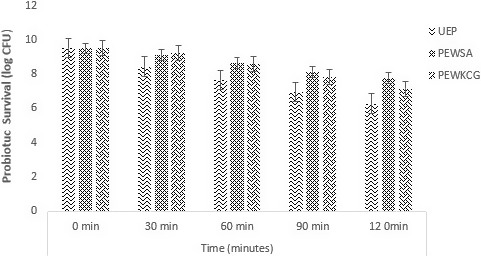
Viability of free and encapsulated (with sodium alginate and carrageenan) probiotics under simulated intestinal conditions during storage intervals (0, 30, 60, 90, and 120 min) compared with control. Each bar represents mean value for viability of treatments. ASWFP (Free/unencapsulated cells), ASWSA (Probiotics encapsulated with sodium alginate), and ASWKC (Probiotics encapsulated with K‐carrageenan)

A rapid reduction in free cells was observed in as compared to the encapsulated probiotics at simulated intestinal fluid pH 7.5 (Figure 6). The encapsulation of the cells with either sodium alginate or carrageenan had statistically significant effect (*p* < .05) on cell survival.

The sodium alginate encapsulated beads showed a log reduction of 1.72 CFU/g while carrageenan coating showed 1.86 CFU/g log reduction. Cell viability in stomach and intestinal conditions is important in order to get the desired benefits of probiotics. The encapsulated probiotic with both sodium alginate and K‐carrageenan showed a significant effect (*p* < .05) on the viability of the cells. The results of the present study are also in accordance with several authors (Atia et al., 2017; Shi et al., 2013) who demonstrated that use of polymer for encapsulation of probiotics protects and maintain the desired viability of probiotics under acidic environment. The encapsulation of the cells with hydrogel materials improves the viability and stability in high pH medium. The survival rate of probiotics in case of sodium alginate was higher as compared to carrageenan. Poor survival in case of unencapsulated cells was recorded. The results demonstrated that encapsulation provides protection to probiotics in simulated gastric conditions. These results are according to the findings of Qi, Liang, Yun, and Guo, (2019), he suggested in his study that encapsulation increases the rate of probiotics viability to 60% in comparison with free cells.

### 3.7 Sensory parameters

**Figure 7:**
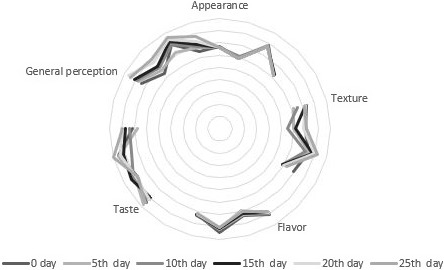
Effect of free (unencapsulated) and encapsulated (with sodium alginate and carrageenan) *Bifidobacterium bifidum* on sensory parameters of dried apple snacks during storage intervals (0, 5, 10, 15, 20, and 25 days) compared with control. Each bar represents mean value of sensory score of treatments. ASC (Control without addition of probiotics), ASWFP (Free/unencapsulated cells), ASWSA (Probiotics encapsulated with sodium alginate), and ASWKC (Probiotics encapsulated with K‐carrageenan)

Results from the sensory evaluation are shown in Figure 7. The results indicated the acceptance of dried apple snack by the evaluation panel. The incorporation of encapsulated probiotic cells has significantly (*p* < .05) affected the sensory properties (appearance, texture, flavor, taste, and general perception) of the product as compared to the control. The sensory panelists did not reject any apple snack sample. The storage period did not affect the sensory attributes of the snacks negatively of the results of the sensory evolution also indicated that the development of apple snack containing probiotic was liked by the sensory panelist and it should be considered as value addition for the food enterprise sector.

## 4 CONCLUSIONS

The development and production of dried apple snacks containing the probiotic microorganism using convectional drying method were successful. Probiotics were added in encapsulated and free form. When stored for 25 days at 4°C, snack with encapsulated bacteria showed more viability than that of free form which indicates the use of dried apple snack as an effective probiotic carrier. The findings of the study indicate that microencapsulation has a key role in improving the viability and stability of probiotics in stressed processing as well as in vitro digestion conditions. Microencapsulation ensured the endorsed level of probiotics under detrimental conditions for health benefits. The results of the study also indicated that the development of snack containing probiotic is an attractive approach to lift human health.

## CONFLICT OF INTEREST

The authors declare that they have no conflict of interest.
